# Development of Nevirapine Resistance in Children Exposed to the Prevention of Mother-to-Child HIV-1 Transmission Programme in Maputo, Mozambique

**DOI:** 10.1371/journal.pone.0131994

**Published:** 2015-07-10

**Authors:** Francisco Antunes, Pereira Zindoga, Perpétua Gomes, Orvalho Augusto, Isabel Mahumane, Luís Veloso, Emília Valadas, Ricardo Camacho

**Affiliations:** 1 Instituto de Saúde Ambiental (ISAMB), Faculdade de Medicina da Universidade de Lisboa, Lisboa, Portugal; 2 Departamento de Medicinas, Instituto Nacional de Saúde, Hospital Central de Maputo, Maputo, Mozambique; 3 Laboratório de Microbiologia Clínica e Biologia Molecular, Serviço de Patologia Clínica, Centro Hospitalar Lisboa Ocidental, Lisboa, Portugal; 4 Saúde da Comunidade, Faculdade de Medicina da Universidade Eduardo Mondlane, Maputo, Mozambique; 5 Plataformas Tecnológicas, Plataforma de Virologia Molecular, Instituto Nacional de Saúde, Maputo, Mozambique; 6 Clinical Data Unit, Eurotrials Scientific Consultants, Lisboa, Portugal; 7 Clínica Universitária de Doenças Infecciosas e Parasitárias, Faculdade de Medicina de Lisboa, Lisboa, Portugal; 8 Department of Microbiology and Immunology, Rega Institute for Medical Research, Leuven, Belgium; University of British Columbia, CANADA

## Abstract

**Background:**

Single-dose nevirapine (sd-NVP) has been the main option for prevention of mother-to-child transmission (PMTCT) of HIV-1 in low-resource settings. However, sd-NVP can induce the selection of HIV-1 resistant mutations in mothers and infants. In Mozambique, there are limited data regarding the profile of NVP resistance associated mutations (RAM) in the context of PMTCT.

**Objectives:**

To assess the prevalence and the factors associated with NVP RAM among children born to HIV-1 infected mothers enrolled in the PMTCT programme adopted in Mozambique.

**Methods:**

One hundred and fifty seven children aged 6 to 48 weeks were sequentially included (July 2011 to March 2012) at four centres in Maputo. Genotyping of RAM was performed in samples with HIV-1 RNA≥ 100 copies/μL (Viroseq). Sequencing was performed with ABI 3100 (Applied Biosystems). Logistic regression modelling was undertaken to identify the factors associated with NVP RAM.

**Results:**

Seventy-nine children had their samples genotyped. Their median age was 7.0 (3–12) months and 92.4% received prophylaxis with sd-NVP at birth plus daily NVP. 35.4% of mothers received antiretrovirals (ARVs) for PMTCT. ARV RAM were detected in 43 (54.4%) of the children. 45.6% of these children had at least one NVP RAM. The most common mutations associated with NVP resistance were K103N (n = 16) and Y181C (n = 15). NVP RAM was significantly associated with mother exposure to PMTCT (crude odds ratio [OR] 30.3, 95% CI 4.93–186.34) and with mother’s CD4 count < 350 cells/mm^3^ (crude OR 3.08, 95% CI 1.02–9.32). In the multivariable analysis the mother’s exposure to PMTCT was the only variable significantly associated with NVP RAM (adjusted OR 48.65, 95% CI 9.33–253.66).

**Conclusions:**

We found a high prevalence of NVP RAM among children who were exposed to the drug regimen for PMTCT in Mozambique. The mothers’ exposure to PMTCT significantly increased the risk of NVP RAM.

## Introduction

In 2010, an estimated 390 000 children were newly infected with HIV, primarily due to mother-to-child transmission (MTCT) [1]. Vertical transmission, which can occur during pregnancy, labour, delivery or breastfeeding, remains the main mode of HIV infection in children [2]. The implementation of preventive health policies such as pregnancy monitoring and the administration of antiretroviral therapies (ART) led to a marked reduction in MTCT rates in high-income countries [3]. However, in limited-resource settings, where full access to ART has not been achieved, MTCT rates remain relatively high [4].

Two of the strategic goals of ‘The Global Plan towards the elimination of new HIV infections among children by 2015 and keeping their mothers alive’ are to achieve an ART coverage among pregnant women of 90% and to reduce MTCT to rates < 5%, in low- or medium-income countries with high estimated prevalence of pregnant women living with HIV [5]. In 2012, the coverage of ART programmes for prevention of MTCT (PMTCT) reached 65% (range: 57 to 70%), among the sub-Saharan African countries [4].

Mozambique is one of the nine countries in sub-Saharan Africa with a prevalence of HIV infection above 10% [6]. In 2009, the coverage of ART in the capital city of Maputo reached 77%. The PMTCT of HIV is a priority to the Mozambican Government, which has implemented a national scale up plan towards the elimination of MTCT up to 2015. However, children’s access to PMTCT programmes in Mozambique is still limited (~59%) [7].

Single-dose nevirapine (sd-NVP), a non-nucleoside reverse transcriptase inhibitor (NNRTI), was adopted by several African countries in the PMTCT due to its good efficacy, low cost, ease of administration and long plasma half-life [8–10]. However, sd-NVP can induce the selection of HIV-1 resistant mutations in mothers and infants. A recent meta-analysis provided a pooled estimate of NVP resistance prevalence of 35.7% and 52.6% in women and children who used sd-NVP, respectively [11]. Some authors suggest that NVP-induced resistance after exposure to MTCT prophylactic programmes may limit virological response to subsequent use of NNRTIs in mothers and infants [3, 12, 13].

The prophylactic regimen adopted by Mozambique follows the Option A preconized by the World Health Organization (WHO) 2009 Guidelines [14]. In infants, this option consist of sd-NVP at birth plus NVP prophylaxis up to the end of breastfeeding. Mothers receive zidovudine (AZT) antepartum, sd-NVP at the onset of labour and twice daily AZT + lamivudine (3TC) for 7 days postpartum (sd-NVP and AZT + 3TC can be omitted if AZT antepartum did not exceed 4 weeks). Surveys conducted in Maputo in 2007 and 2009 revealed rates of transmitted drug resistance to all ART classes below 5% [15].

Thus far, there is limited knowledge regarding the prevalence of NVP viral resistance mutations in children born to HIV-1-infected mothers and who were exposed to maternal or child programs of PMTCT in Mozambique. This knowledge is essential to tailor ARV strategies in this population and maximize the effectiveness of ARV regimens in preventing MTCT.

In our work, we estimated the prevalence of viral resistance to NVP among children exposed to the PMTCT programme adopted in Mozambique. We also explored the most important factors associated with NVP resistance.

## Methods

### Setting and population

This cross-sectional study was carried out between July 2011 and March 2012. Children aged 6 to 48 weeks included in the Mozambican PMTCT program and born to HIV-1 infected mothers were sequentially enrolled in the study according to their scheduled appointment at four paediatric HIV centres in Maputo’s region. The study included mothers who needed ART to treat their own health or mothers who just needed ARVs to reduce the risk of MTCT of HIV.

The PMTCT protocol followed in Mozambique at the time of this study was based on *WHO Rapid Advice—Use of antiretroviral drugs for treating pregnant women and preventing HIV infection in infants–version 2* [14]. HIV-1 infected pregnant women who were eligible for ART (primarily through CD4 screening) started this treatment, irrespective of the gestational age, and continued throughout pregnancy, delivery and thereafter. The first-line ART regimen included AZT + 3TC + NVP. The maternal ARV prophylaxis consisted of antepartum daily AZT as early as 14 weeks of gestation, sd-NVP at onset of labour and twice daily AZT + 3TC for 7 days postpartum.

The prophylaxis in breastfeeding children consisted of sd-NVP at birth and then daily administration of NVP until one week after all exposure to breast milk ended. Non-breastfeeding children received sd-NVP at birth and then daily administration of NVP or AZT until 4 to 6 weeks of age. HIV-infected infants received one of the following ART regimens: AZT or d4T (stavudine) + 3TC + NVP or AZT or d4T + 3TC + LPV/r (lopinavir/ritonavir). Children without CD4 count available and that had received ART out of the PMTCT programme were excluded from the study.

Written informed consent was obtained from all children’s parents or legal guardians.

The study was approved by Mozambique’s National Health Bioethics Committee and by the Mozambican Ministry of Health.

### Data and specimen collection

Socio-demographic and clinical data were obtained from existing medical records. The mother-related data included the WHO clinical stage of disease, type of PMTCT approach (ART or prophylaxis), prophylactic scheme and duration of exposure to ARVs. The mother’s viral load was not assessed at the time of labour (is not routine practice). The child-related variables were gender, age, delivery mode, birth weight, feeding practice, CD4 count, prophylactic regimen and HIV-1 RNA levels.

Two samples of peripheral blood (5 ml each) were collected from each child by venipuncture. One sample was used for routine analysis and the other was shipped within 5 hours to the Immunology Laboratory of Health National Institute in Mozambique. There, 200 μL were used to determine the CD4 count by flow cytometry using FACSCalibur (Becton-Dickinson, San Jose, California, USA); the remaining sample was centrifuged, plasma was isolated and stored at -80°C. This sample was shipped to Hospital Egas Moniz in Lisbon within 12 months after its collection, for genotyping of HIV-1 RNA and detection of ARV resistance associated mutations (RAM). The shipment was performed in leak-proof containers specific for frozen samples and containing dry ice. Temperature was checked at arrival to laboratory and no anomalies were detected. HIV-1 RNA genotyping was performed by means of a commercially available kit (ViroSeq HIV-1 genotyping system; Celera Diagnostics/Abbott Molecular Diagnostics, Rome, Italy) based on RNA extraction, retrotranscription, DNA amplification, and sequencing by an automated sequencer (ABI 3100; Applied Biosystems, Foster City, CA) [16].

Genotyping of ARV RAM was only carried out in samples with HIV-1 RNA levels ≥ 100 copies/μL, using the Stanford Resistance Database (HIVdb version 6.05) [17].

A NVP RAM was considered if at least one of the following genetic changes in reverse transcriptase was detected: A98G, K101E, K101H, K101P, K103N, V106A, V106M, V108I, Y181C, Y181V, Y188L and G190A.

HIV subtypes were determined by phylogenetic analysis of pol region sequences, using the Rega Subtyping Tool v 2.0 [18].

### Statistical Analysis

The sample size was calculated based on the population of Maputo, 1.209.993 persons, and a rate of transmission of HIV drug resistance for all relevant ARVs and ARV classes of 4.0%, observed in Malawi [19], a neighbouring country of Mozambique with similar characteristics (low income, high illiteracy rates, poor sanitation and high rate of sexual activity). A sample of 120 children allowed the estimation of prevalence of NVP RAM with a 95% confidence interval (CI) and a margin of error of 3.5%. Overall, 157 children were enrolled to account for specimen losses above 20%.

Quantitative variables were summarised as median, minimum and maximum and qualitative variables were summarised as absolute frequency. CIs were computed for the most prevalent mutations (> 5 cases). Odds ratio (OR) and 95%CI were used to explore the association between independent variables and NVP RAM.

The bivariable analysis was conducted for all mother and child characteristics potentially associated with NVP RAM. All variables with a *p* value ≤ 0.20 in the bivariable analysis, as well as child’s gender and age, were included in the multivariable logistic regression analysis. Adjusted ORs and 95% CIs were estimated for all independent predictors in the final selected logistic model. All *P* values were two-sided and the level of significance was set at *p* <0.05.

Statistical analysis was performed using the Statistical Package for the Social Sciences, version 19.0 (SPSS Inc., Chicago, IL, USA).

## Results

### General characteristics of the eligible population

Overall, 157 children met the eligibility criteria. The median age of the children was 7.0 months (1–12 months) and 90 (57.3%) were male. Most children (75.8%; 119 of 157) had CD4 percentage >20%. Seventy-one per cent of mothers (111 of 157) were clinical stage 3 of HIV infection and 127 (80.9%) were receiving ART.

Seventy-nine of the 157 children (50.3%) had their sample sequenced for genotypic resistance. The reasons for not performing resistance tests were the absence of HIV-1 RNA results (n = 22) and undetectable HIV-1 RNA levels < 50 copies/μL (n = 56).

Table 1 summarises the characteristics of children with resistance test results as well as the mothers’ variables of interest. The median age of children was 7 months (3–12 months) and 49 of 79 (62.0%) were male. The vast majority of children (87.3%; 69 of 79) were breastfed and CD4 percentage was > 20% in 48 of 79 children (60.8%). ARV prophylaxis consisted of sd-NVP at birth plus daily NVP in 92.4% of the children. Almost all the children had HIV subtype C, 93.6% (74 of 79). The remaining subtypes were A/C, A/D, C/B, C/D and B (data not shown).

**Table 1:** Mother and child socio-demographic and clinical characteristics among the population with resistance test results.

**Children** (n = 79)		
Age (months)	7.0	3–12
Male	49	62.0%
Delivery mode		
vaginal	66	83.5%
caeserian	13	16.5%
Birth weight		
≥ 2.500 g	43	54.4%
< 2.500 g	36	45.6%
Feeding practice		
formula feeding	10	12.7%
exclusive breastfeeding	69	87.3%
CD4 percentage	25.00	2–55%
<10%	10	12.7%
10–20%	21	26.6%
>20%	48	60.8%
ARV prophylaxis		
AZT	6	7.6%
sd-NVP + daily NVP	73	92.4%
**Mother** (n = 79)		
Clinical stage of HIV infection (WHO)		
1 and 2	13	16.5%
3 and 4	66	83.5%
MTCT approach^†^		
ART	51	64.6%
Prophylaxis	28	35.4%
Duration of exposure to ARVs (months)	8.00	0–38
CD4 count (cells/mm^3^)	540.00	108–1120

Data are number (%), median (range).

AZT, zidovudine; sd-NVP, single-dose nevirapine; MTCT, mother-to-child transmission; ARVs, antiretrovirals; ART, antiretroviral therapy; mm^3^, cubic millilitre.

^†^The mother’s first-line ART regimen included AZT + 3TC + NVP. The maternal ARV prophylaxis consisted of antepartum daily AZT as early as 14 weeks of gestation, sd-NVP at onset of labour and twice daily AZT + 3TC for 7 days postpartum.

No statistically significant differences were found for any of the variables of interest between the 79 children who had the samples sequenced and the 78 children who did not.

The mothers were predominantly clinical stage 3 or 4 (83.5%; 66 of 79), 28 (35.4%) received daily AZT as early at 14 weeks of gestation, sd-NVP at onset of labour and twice daily AZT + 3TC for 7 days postpartum, and 51 (64.6%) were on first-line ART regimen. The median duration of mother’s exposure to ARVs was 8 months (0–38 months) (Table 1).

### NVP resistance associated mutations (RAM)

ARV RAM were detected in 43 of the 79 samples that were sequenced (54.4%; 95% CI 43.5–65.4). Thirty-six samples (45.6%; 95% CI 34.6–56.6) had at least one NNTRI RAM, 33 samples (41.8%; 95% CI 30.9–52.65) had NRTI RAM and seven samples (8.9%; 95% CI 2.6–15.1) had PI RAM.

**Fig 1:**
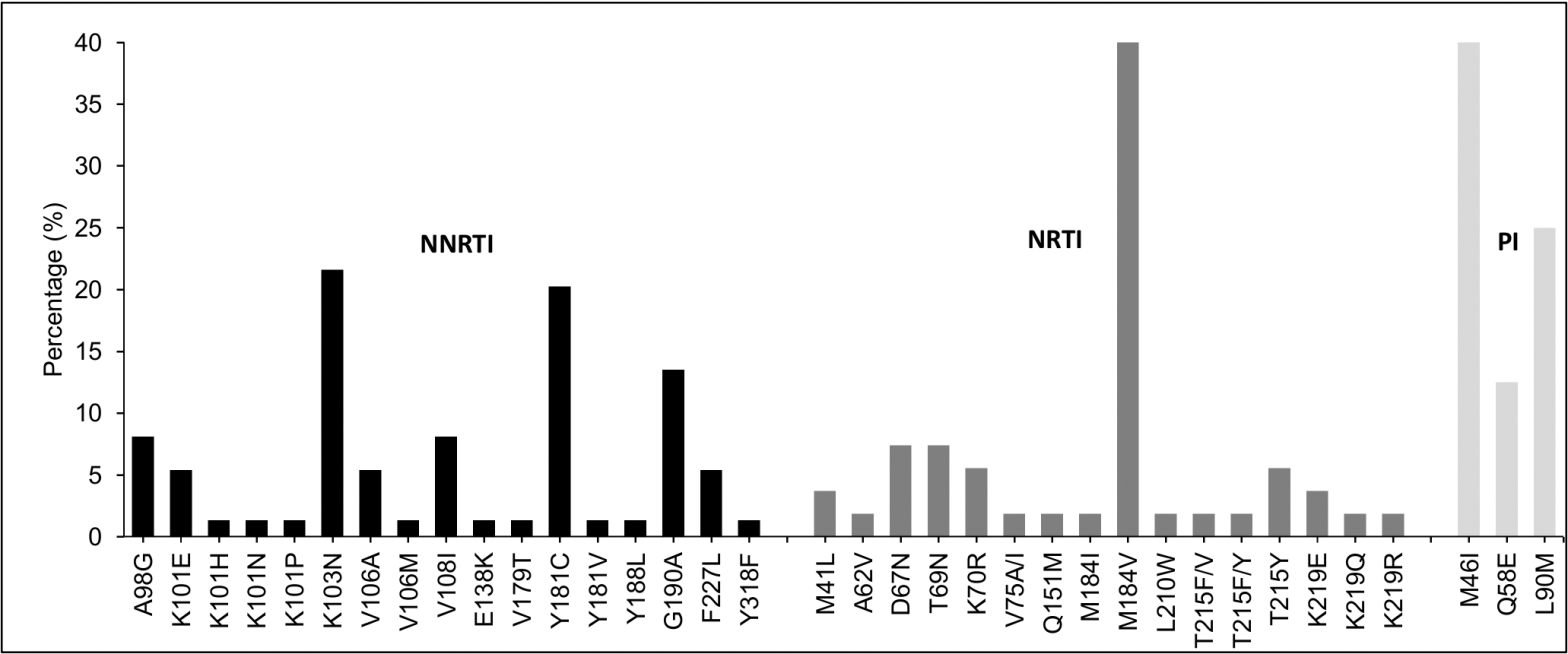
Profile of drug resistance associated mutations by ARV class. Percentages were calculated based on the total number of RAM found for each ARV class (NNTRI = 74, NRTI = 54 and PI = 8).

Overall, 74 NNTRI RAM, 54 NRTI RAM and eight PI RAM were observed. The NNRTI RAM most frequently represented in this ARV class were K103N (n = 16, 21.6%, 95% CI 12.2–31.0) and Y181C (n = 15, 20.3%, 95% CI 11.1–29.4) and G190A (n = 10, 13.5%, 95% CI 5.7–21.3). M184V (n = 27, 50.0%, 95% CI 36.7–63.3), T69N and D67N (n = 4, 7.4%) were the most common NRTI RAM among this therapeutic class. PI resistance mutations were M46I (n = 5, 62.5%) and Q58E (n = 1, 12.5%), L90M (n = 2, 25.0%) (Fig 1).

**Fig 2:**
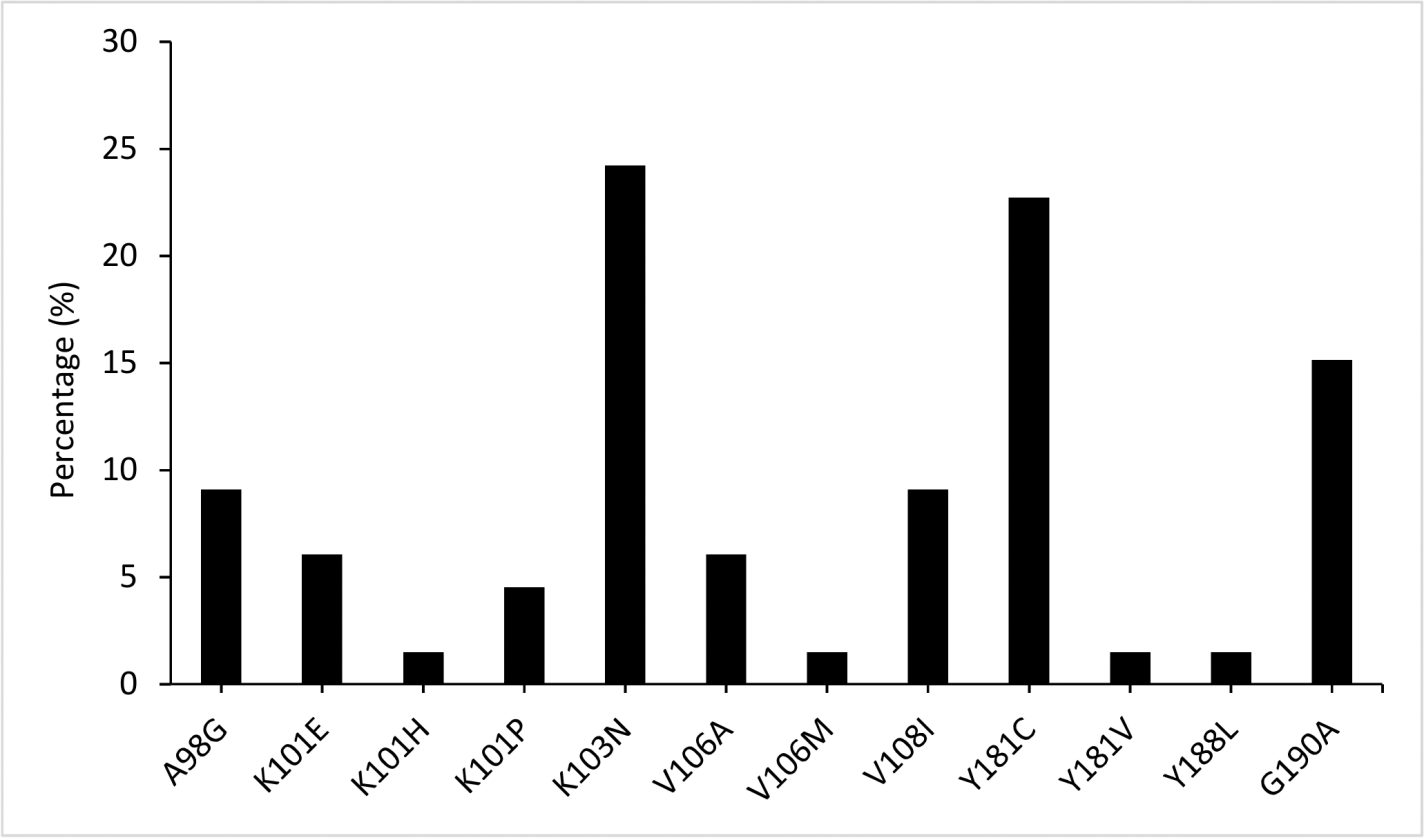
Profile of NVP resistance associated mutations (RAM). Percentages were calculated based on the total number of NVP RAM (n = 66). K103N and Y181C were the most frequent NVP RAM (24.2% and 22.7% of the samples, respectively).

All the samples with NNRTI RAM had at least one NVP RAM. Overall, 66 NVP RAM were found. Of these, the most frequent mutations were K103N (n = 16, 24.2%, 95% CI 13.4–34.6) and Y181C (n = 15, 22.7%, 95% CI 12.6–32.8), and G190A (n = 10, 15.2%, 95% CI 6.5–23.8). There were also multiple occurrences of V108I (n = 6), A98G (n = 6), K101E (n = 4), V106A (n = 4), K101P (n = 1). The mutations K101H, V106M, Y181V and Y188L were found in only one sample each (Fig 2).

### Factors associated with genotypic resistance to NVP

In the bivariable analysis, NVP RAM was significantly associated with mother’s exposure to prophylaxis ARV (crude OR 30.3, 95% CI 4.93–186.34, *P*<0.0001) and with mother’s CD4 count <350 cells/mm^3^ (crude OR 3.08, 95% CI 1.02–9.32, *P*<0.04). No statistically significant association was found between NVP RAM and child-related variables, mother HIV clinical stage and duration of mother’s exposure to ART (Table 2). In the multivariable optimization logistic regression, the mother’s exposure to prophylaxis ARV was the only variable that remained significantly associated with NVP RAM (adjusted OR 48.65, 95% CI 9.33–253.66, *P*<0.001). See S1 Table. Study Dataset.

**Table 2:** Logistic regression predicting NVP RAM adjusted for independent variables of interest.

	NVP RAM (n = 36)		Without NVP RAM (n = 43)		Crude OR (95% CI)	*P-value*	Adjusted OR (95% CI)*	*P-value*
Child gender								
Female	12	40.0%	18	60.0%	reference			
Male	24	48.0%	25	51.0%	1.44 (0.57–3.65)	0.4396	0.88 (0.25–3.12)	0.853
Child age (months)								
< 6	9	36.0%	16	64.0%	reference			
6–12	27	50.0%	27	50.0%	1.78 (0.66–4.79)	0.2482	1.85 (0.50–6.87)	0.356
mean age ± SD	7.80	± 2.89	6.67	± 2.58		0.061		
Delivery mode								
Vaginal	7	53.9%	6	46.2%	reference			
Caeserian	29	43.9%	37	56.1%	0.67 (0.20–2.24)	0.5148		
Birth weight (g)								
< 2500	18	50.0%	18	50.0%	1.39 (0.57–3.42)	0.4690		
≥ 2500	18	41.9%	25	58.1%	reference			
Feeding practice								
Exclusive breastfeeding	33	47.8%	36	52.2%	reference			
Infant formula	3	30.0%	7	70.0%	0.47 (0.11–2.00)	0.2932		
Prophylaxis with NVP in children	32	45.7%	40	57.1%	0.60 (0.12–2.92)	0.5222		
Mother exposure to ARV								
ART	11	21.6%	40	78.4%	reference			
MTCT Prophylaxis	25	89.3%	3	10.7%	**30.3 (4.93–186.34)**	<0.0001	**48.65 (9.33–253.66)**	<0.001
Mother CD4 count (cells/mm^3^)								
< 350	12	66.7%	6	33.3%	**3.08 (1.02–9.32)**	0.0408	1.75 (0.37–8.26)	0.477
≥ 350	24	39.3%	37	60.7%	reference			
mean CD4 count	504.44	± 283.14	619.23	± 214.18		0.043		
Duration of mother’s exposure to ARVs (months)							0.31 (0.08–1.31)	0.111
< 12	8	33.3%	16	66.7%	reference			
≥ 12	28	50.9%	27	49.1%	2.07 (0.75–5.75)	0.1517		
Mother WHO staging of HIV infection								
1 and 2	6	46.2%	7	53.9%	reference			
3 and 4	30	45.5%	36	54.6%	0.97 (0.29–3.23)	0.9633		

Data are number (%); mean (standard deviation).

* The following independent variables were included in the multiple logistic adjusted regression analysis: child gender, child age, mother exposure to ARV, mother CD4 count, maternal duration of exposure to ARVs.

Values in bold denote statistical significance.

ART, antiretroviral therapy; CI, Confidence Interval; OR, Odds Ratio; NVP RAM, nevirapine resistance associated mutations; MTCT, mother-to-child-transmission; mm^3^, cubic millilitre.

## Discussion

Our study aimed to estimate the prevalence of NVP RAM in children born to HIV-1 infected mothers and who were exposed to the PMTCT programme preconized in Mozambique. The prevalence of NVP RAM found in our sample was approximately 46%. This rate is within the broad range of prevalence rates of NVP RAM found among several sub-Saharan African countries, which varies from 27%, in South Africa, to 76% in Malawi [20–22]. In 2010, a prospective study with 740 Mozambican children who had received sd-NVP prophylaxis revealed a high rate of NVP resistance (87%) among the group with established *in utero* infection and a rate of 38% when infection occurred in the *peripartum* period [23].

Of notice, almost all of the children had subtype C HIV infection. This finding is in line with rates previously observed in Mozambique. Several studies showed that both women and infants with subtype C HIV are more likely to develop NVP resistance after exposure to sd-NVP than those infected with other HIV subtypes [24, 25].

We found that the mothers who took AZT and/or sd-NVP to provide MTCT prophylaxis to their infant, instead of combination ART and with a low CD4 count had a statistically significant association with NVP RAM in the bivariable analysis. Children whose mothers had CD4 count < 350 cells/mm^3^, had a higher risk of NVP resistance than those children whose mothers had CD4 count ≥ 350 cells/mm^3^ (OR = 3.08). The low CD4 count observed in mothers could be attributable to virological failure and selection of resistance, which, in turn, could be transmitted to the child. The majority of mothers in our sample (84%) were clinical stage 3 or 4 of HIV infection.

In the multivariable logistic model the mothers that took antiretrovirals to prophylaxis their fetus/infant significantly increased risk of NVP resistance (OR = 48.65) among children comparing to mothers who received ART. This finding corroborates the results observed in Maputo by Vaz et al [26], substantiating the evidence that sd-NVP-based prophylactic regimens lead to a marked increase in the risk of resistance to this ARV and to the subsequent use of NNRTIs [3, 12, 27, 28].

In our study, the risk of viral resistance to NVP was numerically higher among children whose mothers were exposed to ARVs for more than 12 months but this finding had no statistical significance (OR = 2.07).

In our sample, a high proportion of children (87.3%) were breastfed. An analysis of breast milk samples from mothers of city of Beira in Mozambique who were exposed to sd-NVP, showed a persistence of NVP-resistant HIV-1 up to 8 months postpartum [29]. Around 48% of the children in our study that were breastfed had NVP resistance, which is above the rate of NVP resistance detected in Beira, Mozambique (37.5%) [30] and below the rate found in Malawi (66%) [21]. In the bivariable analysis we found no statistically significant association between breastfeeding and an increased risk of NVP RAM.

Thirty-six of the 79 children that were successfully genotyped in our study exhibited at least one mutation conferring resistance to NNRTIs. The most frequent reverse transcriptase mutations were Y181C, K103N and G190A. Similar resistance patterns have been found in other studies [31–33]. PI RAM were detected in a small sample of children, which reflects the low use of PI in this population.

### Limitations

The fact that we did not know the mothers ART history and their ARV resistance profile constitutes one major limitation of our study. As a result, we could not discriminate between resistance mutations that were due to ART or PMTCT and the ones that were selected in the child. WHO endorses the regular surveillance of ARV resistant HIV in patients who receive ART for the first time. Several studies conducted in sub-Saharan African countries found rates of primary resistance around 5% [34]. Furthermore, we were unable to identify if mothers were previously exposed to NVP-containing regimens and, therefore, assess the impact of this potential confounder of NVP resistance development.

We have not investigated the persistence of detectable NVP resistance in our sample. Evidence shows that children’s exposure to sd-NVP is associated with NVP-resistant mutations that can persist for a year or more [35–37]. This persistence seems to be more marked after exposure to extended NVP prophylaxis [38, 39]. Among HIV-infected Ugandan infants with NVP resistance at 6 weeks of age, NVP-resistant HIV variants were still detected at 6 months in all seven infants who received the extended NVP regimen, compared to only one of six infants who received sd-NVP alone [38].

A larger sample would have provided more robust findings regarding the outcomes of interest. Some of the specimens were not sequenced due to poor quality or delay in its shipment. Moreover, the available sequencing assays were not able to detect resistance for samples with HIV RNA levels < 100 copies/μL.

With the rapid scale up of ART in Mozambique, more than 16.000 children have been exposed to ARVs up to 2011. Despite gradually improving access to ART, the proportion of children currently treated in the country is considerably small. As of December 2010 only 23% of HIV-infected children aged < 15 years old in need of ART were receiving this therapy [26, 33].

The identification of the factors most associated with virological response and prevention of resistance to ARVs is crucial for an appropriate management of ART programmes, the optimization of the use of resources and a better definition of treatment strategies. This knowledge is especially relevant in low-income settings, where state of the art techniques such genotyping systems are not widely available.

Recent findings showed that the use of ART including ritonavir-boosted protease inhibitors (PI/r) in PMTCT is more effective than regimens containing one NNRTI [28, 40]. This evidence led to the update of the recommendations followed in Mozambique, which now endorse the use of an ARV regimen that includes PI/r. This measure, accompanied by all the intensive efforts aiming to eliminate AIDS in the paediatric population, may lead to improved outcomes in the PMTCT of HIV.

### Conclusions

This study revealed a high prevalence of NVP resistance associated mutations among children born to HIV-1 infected mothers and who were exposed to the PMTCT programme in Mozambique. In addition, the mothers’ exposure to PMTCT regimen significantly increased the risk of NVP resistance mutations.

Systematic surveillance of ARV associated resistance mutations should be a priority in Mozambique. It is essential to implement and disseminate new recommendations for the PMTCT, which should endorse the introduction of ART to all HIV-infected pregnant women, regardless of CD4 count or the treatment of infected children. The ARV regimens should include one PI/r and should be permanently available in all healthcare units that manage this condition. This is the only way to reduce the incidence of AIDS in this country and improve the survival rates of children living with HIV.

## Supporting Information

S1 Table Study dataset. (XLSX)
